# Supplementary material for: Multi‐dataset identification of innovative feature genes and molecular mechanisms in keratoconus
Source: J Cell Mol Med. 2024 Sep 19;28(18):e70079. doi: 10.1111/jcmm.70079 (PMC11412914; doi:10.1111/jcmm.70079)
Supplement: Supplementary file 2 — Material S2. [file JCMM-28-e70079-s002.zip › 7.GSEA analysis/1.ARL11/KEGG_GLYCOSPHINGOLIPID_BIOSYNTHESIS_GANGLIO_SERIES.html]

Details for gene set KEGG\_GLYCOSPHINGOLIPID\_BIOSYNTHESIS\_GANGLIO\_SERIES[GSEA]

|  || Dataset | ARL11.group.cls#h\_versus\_l |
| Phenotype | group.cls#h\_versus\_l |
| Upregulated in class | h |
| GeneSet | KEGG\_GLYCOSPHINGOLIPID\_BIOSYNTHESIS\_GANGLIO\_SERIES |
| Enrichment Score (ES) | 0.556437 |
| Normalized Enrichment Score (NES) | 1.5347664 |
| Nominal p-value | 0.06412826 |
| FDR q-value | 1.0 |
| FWER p-Value | 0.733 |
Table: GSEA Results Summary

  

Fig 1: Enrichment plot: KEGG\_GLYCOSPHINGOLIPID\_BIOSYNTHESIS\_GANGLIO\_SERIES      
 Profile of the Running ES Score & Positions of GeneSet Members on the Rank Ordered List

  

| SYMBOL | TITLE | RANK IN GENE LIST | RANK METRIC SCORE | RUNNING ES | CORE ENRICHMENT || 1 | ST3GAL5 | na | 1049 | 0.385 | 0.2870 | Yes |
| 2 | SLC33A1 | na | 6371 | 0.127 | 0.2388 | Yes |
| 3 | ST3GAL2 | na | 6403 | 0.126 | 0.3421 | Yes |
| 4 | ST6GALNAC6 | na | 6477 | 0.125 | 0.4429 | Yes |
| 5 | ST6GALNAC5 | na | 7596 | 0.100 | 0.4931 | Yes |
| 6 | ST8SIA5 | na | 9392 | 0.069 | 0.4982 | Yes |
| 7 | ST8SIA1 | na | 10213 | 0.058 | 0.5221 | Yes |
| 8 | B3GALT4 | na | 11248 | 0.045 | 0.5291 | Yes |
| 9 | ST3GAL1 | na | 11491 | 0.042 | 0.5564 | Yes |
| 10 | ST6GALNAC3 | na | 15695 | 0.006 | 0.4403 | No |
| 11 | HEXA | na | 16191 | 0.002 | 0.4280 | No |
| 12 | GLB1 | na | 18269 | -0.012 | 0.3781 | No |
| 13 | B4GALNT1 | na | 20016 | -0.024 | 0.3474 | No |
| 14 | ST6GALNAC4 | na | 22277 | -0.043 | 0.3176 | No |
| 15 | HEXB | na | 22982 | -0.050 | 0.3384 | No |
Table: GSEA details [plain text format]

  

Fig 2: KEGG\_GLYCOSPHINGOLIPID\_BIOSYNTHESIS\_GANGLIO\_SERIES      
 Blue-Pink O' Gram in the Space of the Analyzed GeneSet

  

Fig 3: KEGG\_GLYCOSPHINGOLIPID\_BIOSYNTHESIS\_GANGLIO\_SERIES: Random ES distribution      
 Gene set null distribution of ES for **KEGG\_GLYCOSPHINGOLIPID\_BIOSYNTHESIS\_GANGLIO\_SERIES**
